# Type 2 Autoimmune Pancreatitis in a Young Adult Male: A Diagnostic Challenge

**DOI:** 10.7759/cureus.106881

**Published:** 2026-04-12

**Authors:** Reza Alavi, Syed Hussaini, Juan Gonzalez Velazquez, Faraz Eshaghi, Satish Patel

**Affiliations:** 1 Internal Medicine, HCA Florida Bayonet Point Hospital, Hudson, USA; 2 Gastroenterology, HCA Florida Bayonet Point Hospital, Hudson, USA

**Keywords:** acute pancreatitis, autoimmune hepatitis, autoimmune pancreatitis type 2, endoscopic ultrasound biopsy, granulocytic epithelial lesions, idiopathic duct centric pancreatitis, igg4 negative pancreatitis, immunosuppressive therapy, pancreatic inflammation, young adult pancreatitis

## Abstract

Type 2 autoimmune pancreatitis (AIP), also known as idiopathic duct-centric pancreatitis, is a rare form of chronic pancreatitis that primarily affects younger individuals and is not associated with IgG4-related disease. Unlike type 1 AIP, serum IgG4 levels are typically normal, making diagnosis challenging and often dependent on a combination of clinical, radiologic, histopathologic, and therapeutic response criteria.

We present a 28-year-old male with acute pancreatitis, significant weight loss, and gastrointestinal symptoms. Imaging findings were suggestive of autoimmune pancreatitis. Laboratory evaluation demonstrated elevated total IgG and IgG1 with normal IgG4 levels and a positive anti-smooth muscle antibody. Endoscopic ultrasound-guided pancreatic biopsy revealed nonspecific chronic inflammation and fibrosis, while liver biopsy was consistent with autoimmune hepatitis. In the absence of definitive pancreatic histopathology, the overall clinical presentation was concerning for concomitant type 2 autoimmune pancreatitis.

## Introduction

Autoimmune pancreatitis (AIP) is a distinct form of chronic pancreatitis characterized by immune-mediated pancreatic inflammation and responsiveness to immunosuppressive therapy. Two subtypes have been described with differing clinical, serologic, and histopathologic features [1-3]. Type 1 AIP is associated with IgG4-related disease and commonly presents with elevated serum IgG4 levels and multiorgan involvement [3]. In contrast, type 2 AIP, or idiopathic duct-centric pancreatitis, is a rarer entity that typically affects younger patients, lacks IgG4 elevation, and is not part of the IgG4-related disease spectrum [1,3,4].

Diagnosis of type 2 AIP is often challenging due to nonspecific clinical features and the frequent absence of definitive histopathologic findings. Granulocytic epithelial lesions, characteristic of type 2 AIP, are often patchy and may be missed on biopsy [1,3,5]. As a result, diagnosis typically relies on a combination of clinical presentation, imaging findings, exclusion of alternative etiologies, associated autoimmune conditions, and response to corticosteroid therapy [1,2,4]. We present a case of suspected type 2 AIP in a young adult with biopsy-proven autoimmune hepatitis, highlighting the diagnostic limitations of pancreatic biopsy and the importance of a clinicoradiologic approach.

## Case presentation

A 28-year-old male with a history of autism spectrum disorder presented with acute-onset, severe epigastric abdominal pain, associated with nausea and multiple episodes of green-colored emesis. He also reported intermittent diarrhea over the preceding month and an unintentional 25-pound weight loss. He had a history of recurrent abdominal pain. On admission, he denied alcohol use, tobacco use, or illicit drug use. There was no history of trauma, medication exposure, or family history suggestive of hereditary pancreatitis.

Initial laboratory evaluation demonstrated an elevated lipase level of 351 U/L (>3× the upper limit of normal), consistent with acute pancreatitis. Serum calcium and triglyceride levels were within normal limits. Additional laboratory evaluation revealed elevated total IgG and IgG1 levels with normal IgG4. Autoimmune markers were notable for a positive anti-smooth muscle antibody, while antinuclear antibody and anti-mitochondrial antibody were negative. A summary of laboratory findings is provided in Table 1.

**Table 1:** Laboratory evaluation on presentation Laboratory findings at initial presentation demonstrate elevated lipase, total IgG, IgG1, and anti–smooth muscle antibody, with normal IgG4 levels. These results support an immune-mediated process and help differentiate type 2 autoimmune pancreatitis from IgG4-related disease. (H) indicates values above the reference range.

Test	Result	Reference Range
Lipase (U/L)	351 (H)	13–60
Calcium (mg/dL)	8.7	8.6–10.2
Triglycerides (mg/dL)	126	<150
Total IgG (mg/dL)	2129 (H)	700–1600
IgG1 (mg/dL)	2577 (H)	248–810
IgG2 (mg/dL)	292	130–555
IgG3 (mg/dL)	42	15–102
IgG4 (mg/dL)	18	4–86
IgA (mg/dL)	213	70–400
IgM (mg/dL)	79	40–230
Antinuclear Antibody (ANA)	Negative	Negative
Anti–Smooth Muscle Antibody	118 (H)	<20
Anti-Mitochondrial Antibody	0.39	<1.0
Liver/Kidney Microsomal Antibody	1.3	<2.0

**Figure 1:**
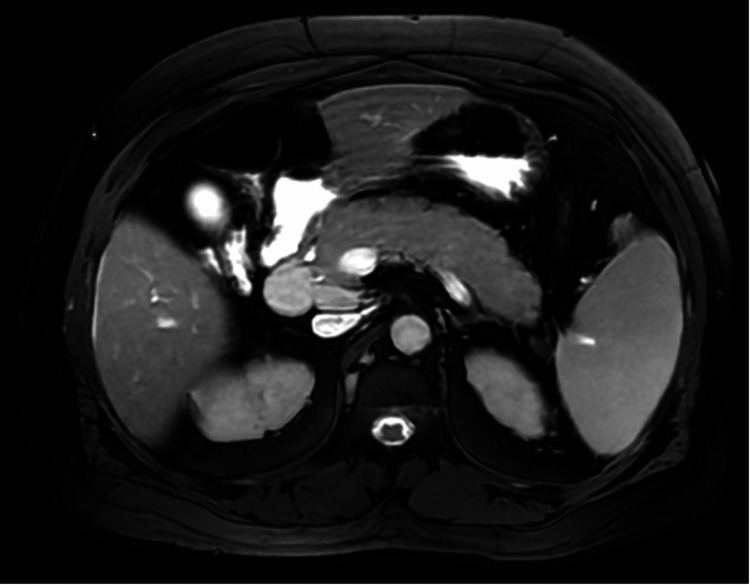
Axial MRCP demonstrating pancreatic enlargement and ductal changes Axial magnetic resonance cholangiopancreatography (MRCP) demonstrates diffuse pancreatic enlargement with loss of normal lobulated contour and mild narrowing of the main pancreatic duct, findings suggestive of autoimmune pancreatitis.

**Figure 2:**
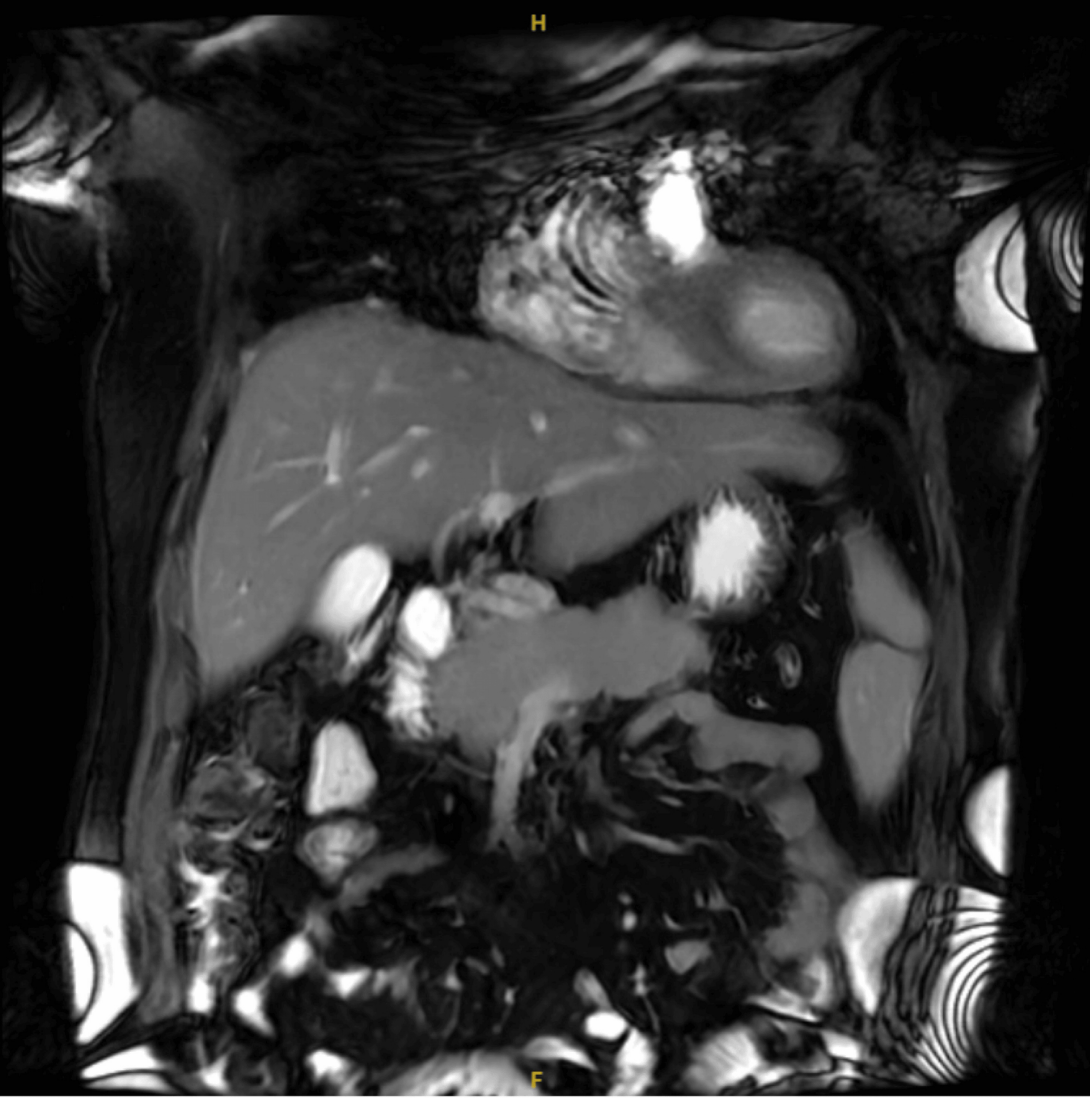
Coronal MRCP showing pancreatic morphology Coronal magnetic resonance cholangiopancreatography (MRCP) image demonstrating diffuse enlargement of the pancreas with relatively homogeneous signal intensity and no focal mass lesion. No significant biliary obstruction is noted.

Liver function tests were significantly elevated (aspartate aminotransferase (AST) 229 U/L, alanine transaminase (ALT) 846 U/L, alkaline phosphatase (ALP) 216 U/L, total bilirubin 0.5 mg/dL) (Table 2). An abdominal ultrasound, obtained as the initial imaging modality, demonstrated mild diffuse pancreatic hypoechogenicity (a nonspecific finding seen in pancreatitis), borderline hepatomegaly, and no biliary ductal dilatation. Given persistent clinical concern and abnormal laboratory findings, magnetic resonance cholangiopancreatography (MRCP) was subsequently performed and demonstrated diffuse pancreatic enlargement with ductal irregularity (Figures 1, 2), without evidence of biliary obstruction or focal mass.

**Table 2:** Liver function tests at presentation (H) indicates values above the reference range. AST: aspartate aminotransferase; ALT: alanine aminotransferase

Test	Value	Reference Range
Total Bilirubin	0.5 mg/dL	0.1–1.2 mg/dL
AST	229 U/L (H)	10–40 U/L
ALT	846 U/L (H)	7–56 U/L
Alkaline Phosphatase	216 U/L (H)	44–147 U/L

Evaluation for common etiologies of pancreatitis, including hypertriglyceridemia, hypercalcemia, medication exposure, and alcohol use, was unremarkable. Viral workup, including hepatitis panel, was negative. Epstein-Barr virus serology was consistent with prior resolved infection (positive EBV IgG). Given the presence of markedly elevated transaminases and positive autoimmune serology, there was concern for autoimmune hepatitis with possible concomitant autoimmune pancreatitis. The patient underwent esophagogastroduodenoscopy with endoscopic ultrasound-guided fine needle biopsy (FNB) of the pancreas, along with liver biopsy. Pancreatic histology demonstrated mild chronic inflammation and focal stromal fibrosis without classic features of autoimmune pancreatitis. Liver biopsy revealed dense portal lymphoplasmacytic infiltrate with interface hepatitis and periportal fibrosis, consistent with autoimmune hepatitis (Table 3).

**Table 3:** Histopathologic findings from pancreatic and liver biopsies FNB: fine-needle biopsy; PAS: periodic acid–Schiff stain; PAS-D: periodic acid–Schiff with diastase; CD138: plasma cell marker; AIP: autoimmune pancreatitis

Specimen	Key Findings	Special Stains / Notes	Interpretation
Pancreas (FNB)	Mild focal chronic inflammation and stromal fibrosis	PAS and trichrome confirm fibrosis	Chronic pancreatitis; no definitive autoimmune pancreatitis
Left Liver Lobe (FNB)	Portal lymphoplasmacytic infiltrate; interface hepatitis; periportal fibrosis	CD138+ plasma cells; PAS-D negative; no iron deposition	Autoimmune hepatitis
Right Liver Lobe (FNB)	Similar findings with plasma cell clusters and mild ductopenia	Trichrome: bridging fibrosis	Autoimmune hepatitis

The patient was initiated on prednisone 20 mg daily and tacrolimus 2 mg twice daily. At the time of submission, treatment had been recently initiated, and clinical and laboratory response is being monitored with planned close outpatient follow-up.

## Discussion

AIP is a rare immune-mediated condition with two distinct subtypes. Type 1 AIP is associated with IgG4-related disease and systemic involvement, whereas type 2 AIP typically affects younger individuals and lacks IgG4 elevation [1-4]. Diagnosis of type 2 AIP remains challenging due to a nonspecific clinical presentation and the limited sensitivity of pancreatic biopsy. Granulocytic epithelial lesions, considered characteristic, are often focal and may not be captured on endoscopic ultrasound-guided biopsy [1,3,5]. Consequently, diagnosis frequently relies on a combination of clinical, radiologic, and serologic findings, exclusion of alternative etiologies, and response to immunosuppressive therapy [1,2,4].

In this case, the patient presented with acute pancreatitis without identifiable common causes. Imaging findings were suggestive of AIP, and serologic evaluation showed elevated total IgG and IgG1 with normal IgG4, supporting a non-IgG4-related process [1-4]. Notably, liver biopsy confirmed autoimmune hepatitis, further supporting an immune-mediated etiology. While autoimmune hepatitis is more commonly associated with type 1 AIP and IgG4-related disease, overlap with type 2 AIP has been described, though less frequently [1,2,6].

The pancreatic biopsy was nondiagnostic, demonstrating only mild chronic inflammation and fibrosis without characteristic granulocytic epithelial lesions. This limitation is well-recognized and highlights the difficulty in establishing a definitive diagnosis when classic histopathologic features are absent [1,3,5]. Given the patient’s clinical presentation with typical abdominal pain, elevated lipase greater than three times the upper limit of normal, supportive imaging findings, serologic profile, associated autoimmune disease, and exclusion of other causes, the overall picture was most consistent with suspected type 2 autoimmune pancreatitis. At the time of submission, corticosteroid therapy had been recently initiated, and the clinical and biochemical response is currently being monitored. A favorable response to steroids may further support the diagnosis; however, the absence of longitudinal follow-up represents a limitation of this report.

In addition, pancreatic pathology and pancreatitis in different patient populations, including older adults, can present with overlapping features that complicate diagnosis [7]. The role of IgG4-related disease in pancreatic inflammation further highlights the spectrum of immune-mediated pancreatobiliary disorders and the importance of distinguishing type 1 from type 2 autoimmune pancreatitis [8]. Furthermore, idiopathic and recurrent acute pancreatitis should remain part of the differential diagnosis in patients with unclear etiology, particularly when initial evaluation is inconclusive [9].

## Conclusions

Type 2 autoimmune pancreatitis is a rare and diagnostically challenging condition, particularly in younger patients without IgG4 elevation or definitive histopathology. This case highlights the limitations of pancreatic biopsy and emphasizes the importance of a comprehensive clinicoradiologic approach integrating clinical presentation, imaging findings, serologic markers, and associated autoimmune disease. In cases where histologic confirmation is lacking, diagnosis often remains presumptive, and response to corticosteroid therapy may provide supportive evidence. Early recognition and appropriate immunosuppressive therapy are essential to prevent disease progression and recurrence.

## References

[1] Li Y, Song H, Meng X (2023). Autoimmune pancreatitis type 2 (idiopathic duct-centric pancreatitis): a comprehensive review. J Autoimmun.

[2] Hart PA, Zen Y, Chari ST (2015). Recent advances in autoimmune pancreatitis. Gastroenterology.

[3] Kamisawa T, Chari ST, Lerch MM, Kim MH, Gress TM, Shimosegawa T (2013). Recent advances in autoimmune pancreatitis: type 1 and type 2. Gut.

[4] de Pretis N, Frulloni L (2020). Autoimmune pancreatitis type 2. Curr Opin Gastroenterol.

[5] Hart PA, Levy MJ, Smyrk TC (2016). Clinical profiles and outcomes in idiopathic duct-centric chronic pancreatitis (type 2 autoimmune pancreatitis): the Mayo Clinic experience. Gut.

[6] Kamisawa T, Zen Y, Pillai S, Stone JH (2015). IgG4-related disease. Lancet.

[7] Faye AS, Kane SV, Calderwood AH (2025). American College of Gastroenterology monograph on geriatrics and GI. Am J Gastroenterol.

[8] Kawa S (2016). Current concepts and diagnosis of IgG4-related pancreatitis (type 1 AIP). Semin Liver Dis.

[9] Guda NM, Trikudanathan G, Freeman ML (2018). Idiopathic recurrent acute pancreatitis. Lancet Gastroenterol Hepatol.

